# Ankylosis of the Shoulder After Traumatic Head Injury: A Late Presentation

**DOI:** 10.7759/cureus.23015

**Published:** 2022-03-10

**Authors:** Mohamed A Elzaki, Mohammed Alamin, Kenneth Kaar, John Kelly

**Affiliations:** 1 Trauma and Orthopedics, University Hospital Galway, Galway, IRL; 2 Orthopedics and Traumatology, Sligo General Hospital, Sligo, IRL

**Keywords:** heterotopic ossification, case report, traumatic brain injury (tbi), shoulder, ankylosis

## Abstract

Heterotopic ossification (HO) is the formation of bone in surrounding soft tissue. In the literature, several causes for this phenomenon were mentioned, trauma - including surgery, burns, and traumatic brain injury. HO in a shoulder is not frequently seen after traumatic brain injury (TBI). This relationship between TBI and HO can be explained in many ways. Surgical treatment entails many complications and important anatomical structures are at risk (e.g., axillary nerve). Surgeon must weigh both, risks and benefits and counsel the patient before taking a decision of surgical excision. We present a rare case of ankylosis of the shoulder following a traumatic brain injury.

## Introduction

Most commonly seen around the hip joint, lamellar bone formation within soft tissue is called heterotopic ossification (HO) [1]. It was seen by Dejerine and Ceillier in 1918 among soldiers sustaining gunshot injuries to the spine around World War I and became paraplegic [2]. Following a precipitating factor, primitive mesenchymal cells in the muscle’s connective tissue septa transform into bone-forming cells [3]. Cadosch et al. suggested presence of humoral influence after traumatic brain injury leading to acceleration of osteoclastic differentiation within muscle tissue [4].

This phenomenon is rarely seen in the shoulder joint after traumatic brain injury (TBI) and may lead to disability and loss of independence as range of motion is diminished. There is evidence that surgical intervention, after maturation, is beneficial [1,5]. We present a case of a completely ankylosed shoulder presented to us three years after head injury that was not amenable for surgery.

## Case presentation

In February 2019, our 29-year-old right-handed male patient went through a rough experience when he was in South Africa. A group of people came into his place of residence and threw him from a balcony of third storey. He sustained multiple injuries, one of which was a traumatic brain injury for which he was offered a craniotomy, followed by an induced coma, and experienced a state of spastic paraplegia. He also lost vision in his right eye and developed heterotrophic ossification in his right shoulder and his left knee manifested by diminished range of motion (ROM).

**Figure 1:**
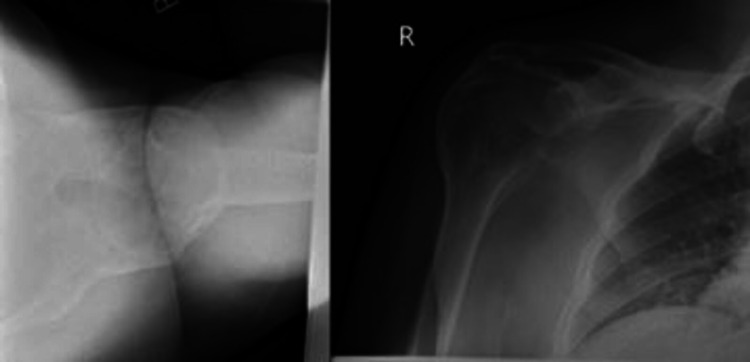
Complete ankylosis of the right shoulder after S-TBI Axillary view (right) and anteroposterior view (left) of the right shoulder showing complete ankylosis after S-TBI. S-TBI: severe traumatic brain injury

Three years later, when he worked as a security guard in Ireland, he presented to the orthopedics outpatient clinic in his county hospital complaining of inability to move his right shoulder. Examination revealed scapulothoracic abduction of 15-30 degrees with no glenohumeral range of motion. There was a sensation at the rudimentary patch area and distal neurovascular status was intact. Full ROM at the elbow was present. He was not able to reach his mouth. On x-ray, the right shoulder is completely ankylosed (Figure 1).

## Discussion

Eight to 20% of patients with closed brain injuries develop HO. Both upper and lower limbs can be affected with the hip being the most affected site (90%), followed by the elbow. Shoulder and knee are not usually seen and wrists, hands, ankles, and feet are even more rarely affected [1].

While metastatic and dystrophic calcifications occur due to trauma and malignancy and are directly related to levels of circulating calcium ion, the development of HO is governed by Chalmers et al.’s three conditions: osteogenic precursor cells, inducing agents, and a permissive environment. All of which found the process of bone healing [6]. Huan et al. included bone morphogenic protein (BMP), thrombin, tumor growth factor-beta 1 (TGF-β1), and vascular endothelial growth factor (VEGF) as osteogenic factors. Calcitonin gene-related peptide (CGRP), substance P, leptin, and melatonin as neuropeptides and hormones regulate the process of osteogenesis. They also included changes in blood-brain barrier permeability, mechanical ventilation, coma and immobilization, and finally inflammation.

**Figure 2:**
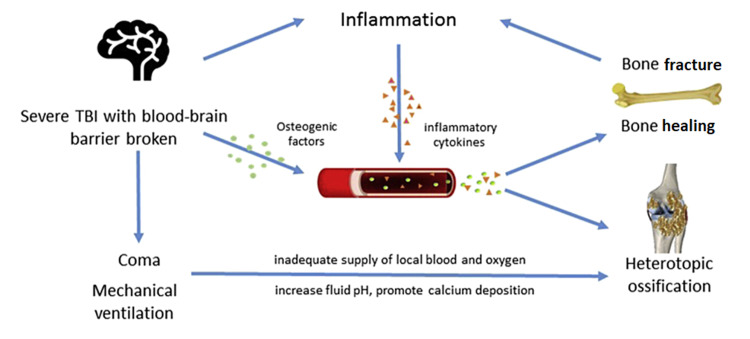
Different factors interact to induce HO Copyright/License Licensee Elsevier (Singapore). This figure is obtained from an open-access article distributed under the terms and conditions of the Creative Commons Attribution-NonCommercial-NoDerivatives 4.0 International (CC BY-NC-ND 4.0). (https://creativecommons.org/licenses/by-nc-nd/4.0/). Source: Huang et al. 2018 [7]. HO: heterotopic ossification; TBI: traumatic brain injury; S-TBI: severe traumatic brain injury.

Figure 2 shows how these factors interact to influence HO formation [7]. Various cytokines are released after brain injury. These, along with the S-TBI and blood-brain barrier damage result in an osteogenic effect. These factors promote healing and increase the risk of HO simultaneously. Also, the enhanced inflammatory response accelerates bone formation. Prolonged mechanical ventilation might alter the chemistry in the body, thereby increasing fluid pH and calcium deposition, and prolonged coma might result in decreased perfusion and oxygenation, promoting HO [7].

Although Dejerine and Ceillier, in 1918, reports on HO in the shoulder following severe traumatic brain injury (S-TBI) were written, not many talked about treatment.​​​​​ Opinions differed regarding surgery and some didn't even mention it. We found that despite a quarter of his patients (265) having TBI, only one case of glenohumeral involvement was reported by Damanski, and surgery was not described [8].​​​​​​ As per the studies by Roberts and Robert and Pankratz, surgery in this region was difficult and risky [9,10]. Warton and Morgan reported 57 shoulders with HO out of 447 TBI patients. All shoulders progressed to ankylosis but there was no mention of surgical intervention [11]. Hoffar et al. treated 11 out of 12 children with shoulder HO and contractures with stretches and resection of new bone [12]. Garland et al. described two ankylosed shoulders out of 27 with HO and no mention of surgery but suggested waiting 1.5 years after TBI [13]. Wenner treated his patient with resection of new bone from the scapulohumeral interval after HO had maturated [14]. ​​​​​An et al. reported a case of peri-articular heterotopic ossification in the shoulder of a 38-year-old woman following head injury and 13 months in a coma. Unusually, a pseudoarthrosis formed within the heterotopic bone. Excision of the heterotopic bone and pseudoarthrosis was performed to improve the range of motion [15]. Keenan and Mehta suggested that in all cases of shoulder HO with range of motion or functional status compromise, the HO should be excised [16].

We are in support of the fact that surgery should be preserved for the cognitively well patient. The surgical aim is to improve mobility and decrease the complications of immobility such as intractable pain and impingement of important neurovascular structures. It also allows for ease of care. We cannot emphasize more on that appropriate preoperative planning is of essence [17].

Due to high incidence of recurrence, a delay in treatment was usually advised until the mass reaches radiological maturity and is silent biologically (alkaline phosphatase levels should normalize and the heterotopic bone should be quiescent on the bone scan). More recent studies, however, advocate early surgery with secondary prophylaxis (i.e., non-steroidal anti-inflammatory drugs {NSAIDs} or irradiation) as it is easier for the surgeon and faster rehabilitation can be achieved [18]. 

This is a complex case, hence counseling the patient on what they want and discussing anticipated risks, and of course outlining the benefits are important. This location had important structures such as the axillary nerve, the axillary artery lying within a bed of muscles that are totally calcified and atrophied. Removal of calcified material will put them in jeopardy. Risk of recurrence is also high; this should be stated as well. Postoperative range of motion was found to be directly proportional to the preoperative range of motion. So, we didn’t expect much of a change in ROM. Wound infection and osteomyelitis are major risks we anticipated as well. The patient should have the big picture clarified and given the choice. 

Our patient chose not to proceed with the surgery after a lengthy discussion. He was unhappy at first as it was his dominant hand and his job needed active hands. He went back to his county to seek further physiotherapy from his local hospital.

## Conclusions

HO is a known complication in orthopedics surgery. It is triggered by several factors including the body response to injury. However, HO with ankylosis of the shoulder after TBI is rare. We presented a case of late presentation after complete ankylosis of the shoulder. We emphasize that surgical treatment should be early, late presentation renders it not always feasible because of anatomical and clinical considerations. In this patient, there was a risk of injuring the shoulder girdle's neurovascular structures. Pros, cons, and anticipated risks and complications were addressed to the patient as patients should always be counseled on the decision of undertaking high-risk surgeries.
